# The Dual Function Model of Attachment Security Priming: Theoretical Framework and Empirical Evidence

**DOI:** 10.3390/ijerph17218093

**Published:** 2020-11-03

**Authors:** Ting Ai, Omri Gillath, Gery C. Karantzas

**Affiliations:** 1 Department of Psychology, University of Kansas, Lawrence, KS 66045, USA; ogillath@ku.edu; 2 Department of Psychology, Deakin University, Melbourne 3125, Australia; gery.karantzas@deakin.edu.au

**Keywords:** attachment security, priming, salivary cortisol, dual function model

## Abstract

According to attachment theory, security providing attachment figures fulfill two main functions: (1) safe haven—providing safety and comfort and reducing stress—helping people regain a sense of security; and (2) secure base—providing resources and a base from which people can spring into action. According to the Dual Function of Security Priming Model, security priming can result in one of two outcomes paralleling these two functions. Which outcome is likely to present itself depends on the level of stress imposed by the context. Here we describe the Dual Function Model of Security Priming (DFSP) Model and provide evidence from a study examining the effects of attachment security priming on hypothalamic-pituitary-adrenal activity. In the study, participants were exposed to security-related cues under high or low/no-stress conditions, while their salivary cortisol concentrations were measured. Cortisol is a suitable index as it is released not only in response to stress, but also more generally when energy needs to be mobilized. We found that while security priming led to significant decreases in salivary cortisol concentrations when presented after a stressor (stress reduction), it led to a significant increase in salivary cortisol concentrations when presented before the stressor (energy mobilization).

## 1. Introduction

Attachment security reflects a set of mental representations in which one perceives the self as competent and worthy of love and support, and others as reliable, trustworthy, willing to meet one’s emotional needs, and provide help and support when needed [1,2,3]. Attachment security develops through interactions with caregivers that are available and responsive during times of need [1,2,3]. Among adults, attachment security is positively associated with various outcomes, such as, relationship satisfaction, the use of constructive coping strategies when facing stress, confidence and a sense of efficacy to pursue personal goals, emotional wellbeing, and personal adjustment [4]. It is therefore no surprise that researchers are interested in understanding how best to enhance attachment security [5].

Although attachment security is often described as a trait-like individual difference, it is thought to be comprised of both trait and state components [6]. Whereas the trait-based component is relatively stable over time, and across situations and relationships, the state-based component reflects aspects of attachment security that are responsive to contextual or environmental cues [7,8]. Thus, a person’s sense of attachment security can temporarily increase or decrease in response to external cues. Researchers are using this fact in their priming studies to manipulate one’s sense of attachment (in)security.

Priming is a well-validated social-cognitive research technique that allows researchers to activate a particular mental representation or association in one’s memory [9,10]. In the attachment domain, researchers often use priming to activate security-enhancing mental representations and examine the outcomes of such activation [11]. Attachment security priming, similar to secure attachment style, has been shown to be associated with a number of positive outcomes, such as, improved affect regulation and increase in pro-relational and pro-social behavior [11]. When examining the types of positive outcomes that are most commonly associated with security priming, it becomes apparent some outcomes can be grouped into two broad categories. The first relates to outcomes that center on stress relief and regaining safety (e.g., improved emotion regulation, increased positive mood, and decreased anxiety and depressive symptoms). The second set of outcomes centers on enhancing mobilization/action (e.g., autonomous exploration and altruistic behaviors) [2,12,13,14]. What might explain why security priming is associated with these two broad outcome types? To answer this question and explain the divide, we proposed the Dual Function Model of Security Priming (DFSP) [15].

### 1.1. The Dual Function Model of Security Priming—Stress Relief versus Mobilization

**Figure 1:**
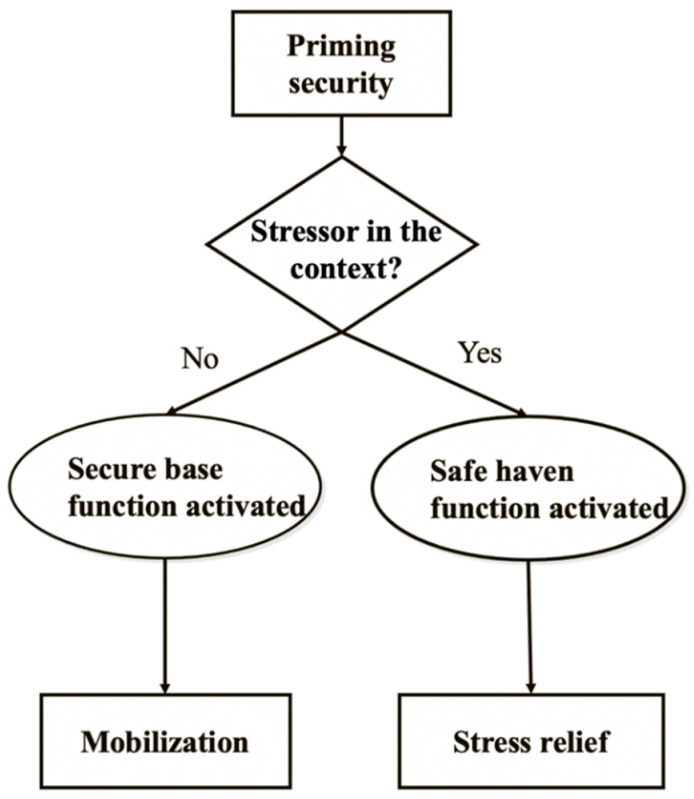
The Dual Function Model of Security Priming.

According to the DFSP model (see Figure 1), exposing people to attachment security cues via attachment-security-related primes can simulate one of two attachment functions—safe haven or secure base [16,17,18,19,20]. A third function of the attachment system—proximity seeking/maintenance—involves regaining closeness to an adult caregiver as a way to increase safety and security through contact. This function is less relevant in adult/adult bonds, as instead of seeking physical proximity adults can summon a mental representation of the caregiver (or of an attachment figure), which simulates the effects of being physically close to the attachment figure. As our model focuses on adults this third function (proximity seeking) is not included in the model.

The safe haven function reflects the provision of support, comfort, reassurance, and relief, whereas the secure base function reflects the facilitation of exploration and the promotion of autonomy. Given that priming should activate feelings of security in a similar way to being in the actual presence of a security-providing attachment figure, one would expect that priming attachment security would activate safe haven, secure base, or both functions (while both could be activated, we argue in our model that often one or the other will be activated, or that the two functions are not activated to the same extent.)

We termed the two aspects of the DFSP (illustrated in Figure 1): (1) stress relief, and (2) mobilization, and they parallel the two functions of safe haven and secure base. Stress relief: when people encounter a threat or stress and they need help, priming security activates an “if-then” script [21]. This script is prototypically comprised of the following: if I encounter an obstacle, then I’ll seek protection from my attachment figure, and s/he will be responsive and supportive. If indeed support is provided, stress should abate, and security should be regained. This script aligns with cognitions associated with the safe haven aspect of security.

Mobilization: in a context in which there is no immediate threat (e.g., the stress level is low or there is no obvious threat), security priming is likely to activate the sense of secure base, and to contribute to what Mikulincer and Shaver [22] refer to as the ‘broaden and build cycle’ of attachment security. They suggest that security increases people’s resilience and expands their skills and capabilities as well as their problem-solving abilities and cognitive flexibility. Priming security under these circumstances activates a different script than the if-then script. This script entails declarative and procedural knowledge that the world is safe; I can call upon attachment figures when I need them. Knowing these things, I can devote attention and effort to personal growth, self-development, and the needs of others. This script is a good depiction of the secure base aspect of security.

The mobilization component of security priming is thought to mobilize energy, and in turn, prepare people to engage in action. There are two routes through which this can occur. The first is on a psychological level. Specifically, security cues may enhance a person’s efficacy and self-evaluations of how much energy s/he has. It is this psychological sense of energy that facilitates active engagement with the environment [23,24]. For example, previous research shows that security primes result in feeling greater subjective energy [25]. The second is on a physiological level, security might activate metabolic processes that prepare the body for action and provide people with the energy to do so [26]. Preliminary evidence for this physiological route comes from studies showing that security priming activates brain areas related to attention and regulation [26], as well as higher blood glucose levels and greater heart rate variability compared to a neutral prime [27].

The DFSP model is similar to Feeney’s (2004) Circle of Security Model in Adulthood. According to Feeney’s model, when a support-recipient (e.g., a child in a strange situation) experiences and perceives life stressors, a support-provider (e.g., a parent) can fulfill the safe haven function by providing help and support that reduces the recipient’s stress. Conversely, when a recipient encounters an exploration opportunity, the provider can engage in secure base behaviors to help the recipient engage in exploration. The Circle of Security in Adulthood Model specifically highlights support dynamics in romantic relationships. The Dual Function model of security priming builds and extends Feeney’s model, suggesting that security priming acts in a similar way to a security-providing relationship partner, and either relieving stress or encouraging mobilization, depending on the circumstances (The DFSP is not only focusing on romantic relationships or on help provision).

Both the Dual Function Model we propose and Feeney’s [16] Circle of Security in Adulthood Model suggest that the functions of attachment priming (or attachment figures) change in line with the context, specifically the level of stress imposed by the social context, and how this stress is appraised by the individual. This idea is consistent with the stress coping theory which suggested that people appraise environmental context as either a threat or a challenge [28,29]. When environmental demands are perceived as exceeding one’s resources or ability to cope, people feel threatened and respond with high negative affect and an inadequate or disorganized mobilization of resources. Conversely, when environmental demands are appraised as being within one’s resources or ability to cope, people feel challenged and respond with positive affect and efficient organized mobilization of resources. Applying Tomaka et al. [29]’s ideas to attachment: when people feel threatened, security priming generally functions to relieve negative affect and restore physiological and psychological functioning (safe haven/stress relief). Conversely, when people feel challenged (that is, when levels of stress/demands are lower such that they can be managed), security priming generally functions to facilitate the mobilization of resources and engagement with the challenge (secure base/mobilization).

The DFSP model highlights the need to take the effects of context into consideration when examining the outcomes of attachment security priming. This is in line with the resource computation model proposed by Cesario and Jonas [30]. According to the resource computation model, social behaviors following priming should be understood as the output of a computational process that assesses what a person can and cannot accomplish in response to the prime. According to our model, when people are exposed to a security prime, a computational process is initiated. Specifically, people assess the resources available to them at that moment before executing their responses. People execute different behavioral responses depending on that process and the assessment of resources. In a high stress context, people are likely to feel the environment presents challenges more difficult than they can handle and they do not have enough resources to focus on others, so they may avoid action and focus inwardly on the self and on stress reduction. Conversely, in a low stress context, people are likely to assess they have enough resources to interact with the environment and to dedicate to others (e.g., alleviate others’ pain), and thus can focus on the outside world (exploring the environment), or on others (helping people in need).

### 1.2. Effects of Attachment Security Priming on Salivary Cortisol Concentration

One way to test the DFSP is by examining people’s physiological reactions, specifically, hypothalamic-pituitary-adrenal (HPA) activity following exposure to security priming. Physiological systems regulate internal processes in response to external events [31]. In the face of stressors, all physiological systems involved with mobilization of recourses (e.g., sympathetic nervous system (SNS) and HPA) are activated while other systems that are not involved (e.g., digestive system) are being deactivated. The HPA system is usually regarded as one of the main physiological systems that guide responses to stressors [31].

An alternative perspective argues that the HPA system serves more generally as a metabolic sensory system that monitors external events and initiates the subsequent mobilization of energy if needed [31,32]. Cannon [33] coined the phrase ‘fight or flight’, an emergency metabolic state underlying the preparation for sudden action, which involves the anticipatory activation of metabolic processes. Fight-or-flight response represents the initial stage (alarm) of the body’s physiological reaction to external events [34] and includes the activation of the HPA system [34]. Following the activation of the HPA system, the glucocorticoids in the body significantly increase and reach their peak response. Glucocorticoids during this stage serve multiple functions including freeing stored energy in the form of glucose and preserving energy for the brain [35].

The activation of the HPA system initiates a predicable temporal sequence including the release of glucocorticoids [31]. The activation of the HPA system stimulates the corticotropin releasing hormone (CRH) secretion from the hypothalamus. CRH then stimulates adrenocorticotropin hormone (ACTH) secretion from the pituitary, and finally ACTH stimulates the secretion of cortisol, a hormone that alters the function of numerous tissues in order to mobilize or store energy to meet the demands associated with external events [36]. Cortisol then exerts negative feedback on the hypothalamus and pituitary gland, which inhibits further activation of the HPA system and results in cortisol concentrations returning to baseline [31]. The negative feedback loop is essential to restore physiological homeostasis. If the HPA response is exaggerated or continues over an extended period, cortisol can have detrimental outcomes on somatic health and cognitive functioning, such as inflammatory and immune diseases and metabolic difficulties [37].

Attachment processes have been shown in multiple studies to be associated with the activation of the HPA system [38,39,40,41]. Most of these studies focused on the associations between attachment style and cortisol responses to acute stress or awakening cortisol. For example, Powers et al. [40] demonstrated that insecure individuals (both anxiously and avoidantly attached individuals) exhibited greater cortisol reaction in response to stressors such as relationship conflict than secure ones. Quirin and colleagues [41] found that individuals low on attachment anxiety showed a lower salivary cortisol response to acute stressors but higher cortisol concentrations when awakening—as compared with those who were high on attachment anxiety. Some researchers conceptualize the awakening response as a cortisol mobilization response during which the body mobilizes its resources for the day [42]. Thus, it is reasonable to suggest that individuals high on attachment security are more prepared for action to engage with the environment than their insecure counterparts when they wake up.

### 1.3. Current Study

In the current study, we examined salivary cortisol response following exposure to security primes, either before or after exposure to a stressor. Half of the participants were randomly assigned to a condition where security priming was presented before the stressor, and the other half was assigned to the condition where security priming was presented after the stressor. We hypothesized that: (1) being exposed to an attachment security prime after exposure to a stressor (threats of romantic break-up) will result in a decrease in salivary cortisol concentrations back to baseline—supporting the stress relief component of our model; whereas (2) being exposed to an attachment security priming before a stressor will result in an increase in salivary cortisol concentrations—representing readiness for action—supporting the mobilization component of our model.

## 2. Materials and Methods

### 2.1. Participants

Seventy-four undergraduates (44 women and 30 men, aged 18–24, mean age = 20 years) who had experienced at least one painful relationship breakup were recruited from a large university in California, the United States. Fifty-eight percent were romantically involved at the time of the experiment. Thirty-five percent of the sample classified themselves as Asian; 35% as Caucasian; 14% as Hispanic; 3% as African-American; and 13% as “mixed” or “other”. Participants received credits in an introductory psychology course for participating. The study was approved by the Institutional Review Board of the university.

### 2.2. Procedure

Participants were tested individually in a 90-min session presented as a study of “physiological reactions to relationship-related memories”. Participants were asked to refrain from eating, drinking, or brushing their teeth in the two hours before the study; to avoid consuming caffeine 12 h before the study; and to avoid exercising or drinking alcohol 24 h before the study. All participants were scheduled to appear between 4:00 and 7:00 P.M., when cortisol levels were expected to be relatively low and stable. Participants first consented and then completed a short demographic questionnaire and an adult attachment measure.

Participants next went through four stages: In the first stage, they listened to relaxing music through headphones for 5 min to establish “baseline” levels. The next two stages were a positive and a negative interview (15 min on average for each interview). Participants were interviewed in person by a trained research assistant. Each interview included 16 questions. The first 4 questions were warm-up questions, followed by 3 questions dealing with the event (breakup in the negative interview: e.g., “*What led up to the breakup, was it something that you did or something you didn’t do?*” or a support scenario in the positive event: e.g., “*What led up to the situation in which an important relationship partner loved, helped, appreciated, or supported and benefited you in an important way?*”) The remaining 9 questions addressed repercussions of the event and how the participant currently felt about it (e.g., “*Do you still have any resentment or animosity toward your former partner?*” in the negative interview; “*Do you still feel grateful or love toward that person?*” in the positive interview). The relationship breakup interview was intended to evoke mostly negative feelings associated with attachment insecurity and relationship loss, and hence, to act as a stressor. Conversely, the relationship support interview was intended to evoke mostly positive emotions, such as remembering feeling loved and accepted, to prime attachment security.

The order of the two interviews was counterbalanced across participants. About half of the participants were first exposed to the security prime (positive/support interview) and then to the stressor (negative/breakup interview; the relief condition); whereas the other half were first exposed to the stressor and then to the security prime (the mobilization condition). There was a 5-min break between the two interviews, during which research assistants could collect the saliva samples from participants. Given that cortisol takes 10–15 min to manifest in saliva, saliva was collected before the interviews began (at the end of the baseline period), again after each of the two interviews, and 20 min after the second interview ended. In the last 20 min (i.e., post the second interview), participants completed questionnaires, such as the Big Five inventory [43]. At the end of each experimental session (after the fourth sample was collected), saliva samples were kept frozen at −20 °C until assay.

### 2.3. Measures

Adult attachment styles were assessed using the Experiences in Close Relationships scale (ECR) [44], a 36-item self-report questionnaire containing two subscales (anxiety: α = 0.92, mean = 3.97, SD = 1.12; and avoidance: α = 0.94, mean = 2.90, SD = 0.94). The two scale scores were slightly correlated in this study (*r* = 0.11, *p* < 0.05). High scores on each dimension reflect attachment anxiety and avoidance respectively. Low scores on both dimensions reflect attachment security. Because attachment insecurity, especially anxiety, is often associated with neuroticism [45], we wanted to make sure that any effects we obtained were specifically related to attachment insecurities and not to these more general personality traits. We, therefore, included the neuroticism scale from the Big Five Inventory (BFI, α = 0.84) [43] to evaluate and control for its effect.

Cortisol concentrations were determined from saliva samples, a reliable and valid method of assessing cortisol [46]. Salivary cortisol concentrations show the same dimensions of the stress response as plasma cortisol concentrations [47]. The experimenters obtained 7.5 milliliters of saliva for each assessment by asking participants to chew a piece of Trident^®^ Original Flavor sugar-free gum and then drool saliva directly into a Nunc cryotube. Prior to assay, the samples were thawed, centrifuged at 3000 rpm for 10 min to separate the aqueous component from mucins and other suspended particles. Cortisol concentrations were estimated in duplicate using commercial radioimmunoassay kits (Diagnostics Products Corp., Los Angeles, CA, USA). Assay procedures were modified to accommodate overall lower concentrations of cortisol in human saliva relative to plasma as follows: (a) standards were diluted to concentrations ranging from 2.76 to 345 nmol/L; (b) sample volume was increased to 200 µL; and (c) incubation time was extended to 3 h. Serial dilution of samples indicated that the modified assay displayed a linearity of 0.98 and a least detectable dose of 0.548 nmol/L. Intra- and inter-assay coefficients of variation were 2.56 and 14.30, respectively.

## 3. Results

**Figure 2:**
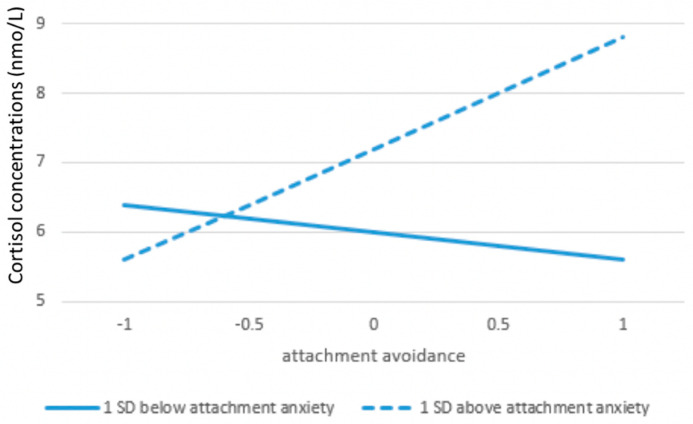
The relationship between baseline salivary cortisol concentrations and attachment avoidance for high and low attachment anxiety.

Preliminary analysis predicting baseline cortisol concentrations from attachment anxiety and avoidance yielded no main effects, but there was a significant interaction between attachment anxiety and avoidance, *β* = 0.29, t (70) = 2.31, *p* < 0.05. Probing the interaction revealed that the slope of avoidance was significantly positive when anxiety was one SD above the mean (*b* = 1.63, *p* < 0.05), but not different from zero when anxiety was one SD below the mean (*b* = −0.35, ns). Thus, participants who were high on both the anxiety and avoidance dimensions (the group Bartholomew and Horowitz, 1991, labeled fearful avoidants) had higher baseline salivary cortisol concentrations see Figure 2 [41,48].

**Figure 3:**
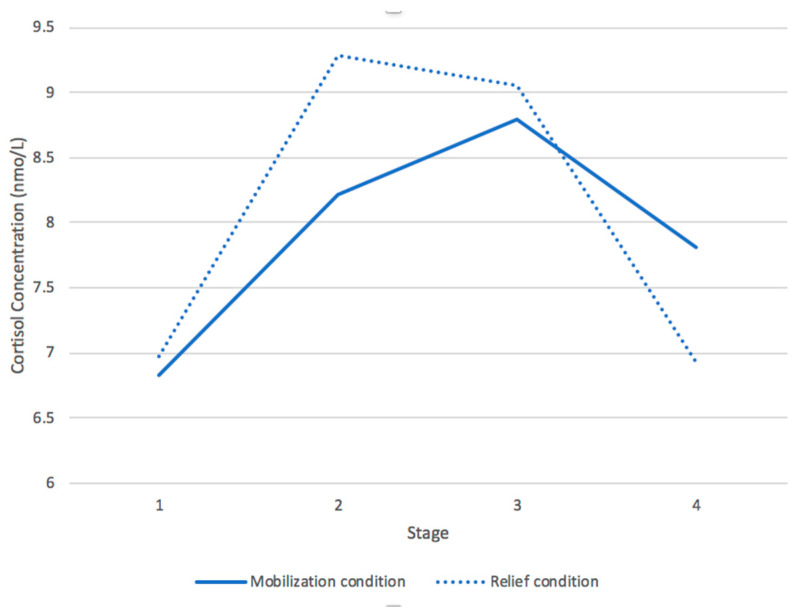
The change of salivary cortisol concentrations throughout four stages in two conditions.

Because the cortisol response is known to have both a reactivity and a recovery phase [increasing from baseline in response to a stressor and then decreasing back to baseline; see, e.g., [40], we used linear growth modeling to examine the linear and quadratic associations between task stages and cortisol concentrations. Both the linear *β* = 0.08, *t* (64) = 3.01, *p* < 0.001 and the quadratic *β* = −0.002, *t* (64) = −4.25, *p* < 0.001, effects were significant (see Table 1 for means and SDs and Figure 3 for the overall patterns for the two conditions). These findings suggest that changes in cortisol concentrations can be best represented by two line segments—the first (Seg 1) representing the reactivity or the increase phase, and the second (Seg 2) representing the recovery phase or the decrease back toward baseline. Seg 1 represents the changes in cortisol concentrations between the first and second measurements (baseline and end of interview one), and Seg 2 represents the changes between the third and last measurements (end of the 2nd interview and completion of the survey).

To analyze the interaction with attachment, we used a spline regression model [49] where the peak point was located at the transition from the first interview to the second interview. In the model, we included Seg 1, Seg 2, mean-centered attachment scores, condition (security priming first or acute stressor first), and gender. (As our analysis yielded a main effect of gender, *β* = 2.18, *t* (48) = 2.18, *p* < 0.05, with women having higher mean cortisol concentrations than men, we included gender in the main analysis. Gender, however, did not interact with any other independent variables). We also entered neuroticism as a control. We used the four salivary cortisol concentration measures (one from each stage) as the data points. The analysis only yielded a significant effect for Seg 1, *β* = 0.10, *t* (152) = 2.29, *p* < 0.05, which indicated that cortisol concentrations increased in Seg 1 regardless of condition. To examine whether cortisol concentrations increased after the security priming in the mobilization condition, we divided the file by condition and then re-ran the spline model analyses. In the mobilization condition, cortisol concentrations increased after exposure to a security prime, *β* = 0.12, *t* (90) = 3.16, *p* < 0.05. As expected, in a safe environment security led to an increase in cortisol, reflecting people getting ready for action. As one would expect, in the relief condition, cortisol concentrations also increased, assumingly due to the exposure to a stressor, *β* = 0.09, *t* (62) = 3.02, *p* < 0.05.

**Figure 4:**
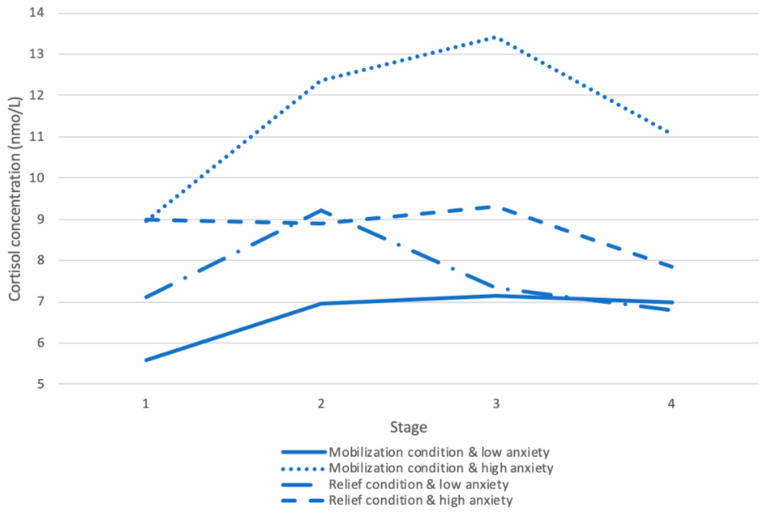
Changes in salivary cortisol concentrations throughout the four stages among people high and low on attachment anxiety.

The analysis also yielded a significant effect for Seg 2, *β* = −0.07, *t* (152) = −2.78, *p* < 0.01; and a three-way interaction between Seg 2, condition, and attachment anxiety, *β* = −0.08, *t* (152) = −2.42, *p* < 0.05. To examine the nature of the interaction between Seg 2, condition, and attachment anxiety, we divided the file by condition and then re-ran the spline model analyses. In the mobilization condition, anxiety was positively related to peak cortisol concentration, *β* = 3.50, *t* (30) = 4.73, *p* < 0.001, but negatively related to the recovery, *β* = −0.06, *t* (90) = −2.30, *p* < 0.05. These results suggest that people high on attachment anxiety, exhibited higher peak cortisol, and slower return of cortisol levels to baseline concentrations. People low on anxiety or in the relief condition did not show these patterns (see Figure 4 for the overall patterns for the high attachment anxiety and low attachment anxiety). There were no significant effects of anxiety on cortisol concentration in the relief condition. These findings indicate that regardless of attachment style, relationship-related stress generally caused a significant increase in salivary cortisol concentrations. Additionally, security priming after the stressor generally caused a significant decrease in cortisol concentrations regardless of attachment scores. (Please refer to the supplementary materials for additional analyses).

## 4. Discussion

The main goal of the current study was to provide evidence in support of the Dual Function model of Security Priming. We achieved this goal by examining the salivary cortisol response following exposure to security primes, either before or after exposure to a stressor. Supporting our hypotheses, exposure to a security priming after an acute stressor resulted in a decrease in salivary cortisol concentrations—reflecting that security priming facilitated stress relief. Conversely, exposure to a security prime before an acute stressor resulted in an increase in salivary cortisol concentrations—reflecting that security priming facilitated the mobilization of energy.

Cortisol, as one of the main glucocorticoids released by the HPA system, mobilizes energy resources to provide “fuel” for the body and modulates other physiological systems to effectively respond to environmental challenges [37]. Although the HPA axis is often considered a system that activates in response to stress, an alternative perspective argues that cortisol is released to mobilize energy more generally [31]. For example, López, Hay, and Conklin [50] found that women experienced a significant increase in both testosterone and cortisol following exposure to images of attractive men. They suggested that increased cortisol may mobilize energy to prepare the body to deal with courtship attempts.

In the current study, we demonstrated that priming attachment security before the stressor activated HPA axis and resulted in increased cortisol, potentially helping to mobilize energy to prepare people for the challenge they are facing. These findings support the mobilization function of the DFSP Model. When the level of stress imposed by the context was low, priming attachment security simulated a secure base response, preparing individuals for action by increasing cortisol concentrations and mobilizing energy. Thus, in a low stress context, an increased sense of attachment security signals to the individual that the world is generally safe, that attachment figures are available if called upon, and that it is possible to explore the environment curiously and confidently. The mobilization process is physiologically manifested as the activation of the HPA system and increased cortisol levels. The increase in cortisol is then expected to free stored energy in the form of glucose and to preserve energy for the brain. Indeed, in an unpublished study from our lab, we found security priming resulted in higher salivary glucose levels compared with neutral control primes [51].

Supporting the stress relief function of the DFSP model, we found that being exposed to a security prime in the high stress condition reduced salivary cortisol concentrations. Priming security in this context simulates the safe haven function, thus alleviating stress and facilitating stress recovery [46]. When experiencing adversity, security priming effectively buffers the HPA stress response by providing a script of how to respond and facilitating the use of constructive strategies to regulate stress [21]. These cognitive and affective processes relieve stress and alleviate HPA activity [40].

We also found that in the mobilization condition, being high on attachment anxiety was positively associated with salivary cortisol concentrations following exposure to security primes, and with slower recovery back to the baseline after the stressor. A potential explanation for this finding is that among individuals high on attachment anxiety, activating attachment security might simultaneously activate a sense of security and anxiety of being abandoned [52]. This, in turn, may cause extra stress among anxiously attached individuals. As Mikulincer et al. [52] showed, individuals high on attachment anxiety exhibit strong relational ambivalence. They desire closeness and security, while at the same time, fear abandonment and rejection. The slower recovery following the stress among anxiously attached individuals may have been due to their difficulty controlling their emotions and negative thoughts, or, their tendency to ruminate on negative memories [53].

### 4.1. Limitations and Future Directions

Overall, the evidence we provide here is preliminary, and our conclusions should be taken with caution. There is a need for future studies to use other primes (e.g., words), use different control conditions (e.g., positive affect), find other ways to manipulate threat vs. challenge, and other ways to assess the tendency for mobilization vs. relaxation, such as self-reports and other physiological measure (e.g., heart rate, vagal tone).

Future studies can also help with addressing the limitations of the current study. First, we did not include a control group. Comparing the change of salivary cortisol concentrations between two conditions, we found that both exposure to a stressor and to a security prime significantly increased salivary cortisol concentrations. It suggests that both stress and security priming resulted in the activation of the HPA system to mobilize resources. However, a control group would have allowed us to capture a fuller picture of the changes in cortisol levels. Specifically it would have allowed us to not only have a baseline for comparison, but also rule out the possibility that the increase of salivary cortisol concentrations was due to factors other than the manipulation (e.g., being seated in the lab, communicating with research assistants).

Another related limitation has to do with the lack of psychological measures of stress and mobilization. Salivary cortisol increase can represent either physiological stress or mobilization response. We concluded that the increase in salivary cortisol following the stressor reflected a stress response; whereas the increase following the security prime reflected a mobilization response. It is possible, however, that people also exhibited stress response following the security prime. In future studies, researchers should include psychological measures alongside the physiological measures to examine whether one’s subjective experience (psychological stress or mobilization) corresponds with their physiological responses. Besides the lack of psychological measures of stress and mobilization, we also did not include any measures assessing the severity of the breakup that participants recalled. It is possible that the negative affect generated by recalling the breakup varied between people, and therefore induced different levels of stress. Future studies should control for this possible variation or use different stressors (e.g., speech tasks).

Third, our findings do not rule out the alternative explanation that the increase in salivary cortisol following exposure to security primes may have been due to excitement or arousal [36]. Future studies should include positive non-attachment-related primes as a comparison to rule out such alternative explanations. For example, the use of an interview as the security prime in the current study could have resulted in increased stress due to the need to self-disclose, which in turn may have resulted in an increase in cortisol (as a stress response).

It should also be noted that the sample for the current study was ethnically diverse. However, the proportion of Asian participants in our sample may have contributed to the findings that cortisol increased after the positive interview. Research shows that Asian people often report feeling uncomfortable when receiving support [54]. Future research should test other kinds of security primes (e.g., presenting the word ‘love’ or an image of a mother embracing her baby) to eliminate confounds that were potentially created by the interview.

### 4.2. Implications of DFSP Model

Despite these limitations, the current study provides preliminary evidence to support the DFSP Model. By integrating extensive theory and research, the DFSP Model contributes to the literature and has important implications for future research on attachment theory. First, the DFSP Model responds to rising doubts about the replicability of priming effects. Priming effects are not only a non-motivational and direct expression of activated mental representations, but a computational process that incorporates information from multiple sources [30]. Whether a security priming effect can be replicated may depend not only on the priming itself (e.g., subliminal or supraliminal), but also on the input from the environment. The DFSP Model identifies the stress imposed by the context as an input that participants are likely to process, and in turn, the stress influences priming effects. Future research may explore other inputs (e.g., participants’ current bodily states, goals, and the presence of others) that may also influence the effects of security priming.

Second, the DFSP Model suggests pathways and mediating process through which security priming can lead to enhanced attachment security. Security priming has been demonstrated as an effective way to boost people’s attachment security [11,13]. What is still unclear to researchers is the process through which this boost occurs. The DFSP Model suggests that security priming may enhance people’s attachment security through both secure base and safe haven functions. Future research may further test whether both pathways effectively contribute to enhanced attachment security, and whether people’s attachment styles moderate the effectiveness of the two pathways. For example, is the safe haven pathway more effective for people high on attachment anxiety?

Third, the DFSP Model suggests directions for future research on security priming effects. Security priming can function both as a safe haven to buffer stress and as a secure base to mobilize energy. As reviewed in the introduction, previous research has examined the effects of security priming on some aspects of these two functions, but a lot more work needs to be done. For example, only a few studies have examined the effects of security priming on exploration behaviors [55]. Thus, future research should investigate how the two paths of security priming influence people’s academic achievements, coping with challenges, and personal growth. Future research may also explore the physiological underpinnings of these effects.

## 5. Conclusions

The goal of this article was to explore the new DFSP Model, according to which security priming can simulate safe haven or secure base functions, depending on the circumstances. The model helps explain the duality in the security priming literature. After introducing the model, we reviewed the relevant literature and provided preliminary evidence to support the model by showing that security priming increased salivary cortisol concentrations in a low stress level context but decreased cortisol levels in a high stress level context. With further empirical support the model could provide a framework for improving the understanding of the diverse effects of security priming.

## Figures and Tables

**Table 1:** Descriptive Statistics of Cortisol Concentrations (nmo/L) Under Four Blocks in Two Conditions.

	Mobilization			Relief				
	Time 1 Baseline	Time 2	Time 3	Time 4	Time 1 Baseline	Time 2	Time 3	Time 4
Mean	6.821	8.212	8.791	7.810	6.977	9.279	9.045	6.923
SD	4.445	5.850	4.834	3.861	3.688	5.881	5.121	3.400

## Supplementary Materials

The following are available online at https://www.mdpi.com/1660-4601/17/21/8093/s1.
