# Supplementary material for: The Dual Function Model of Attachment Security Priming: Theoretical Framework and Empirical Evidence
Source: Int J Environ Res Public Health. 2020 Nov 3;17(21):8093. doi: 10.3390/ijerph17218093 (PMC7663451; doi:10.3390/ijerph17218093)
Supplement: Supplementary file 1 [file ijerph-17-08093-s001.pdf]

Model 1: Splines with Attachment and condition and their interactions and neuroticism on Both Slop

Solution for Fixed Effects

Effect	Estimate	Standard Error	DF	t Value	Pr > |t|
Intercept	7.9387	2.2417	48	3.54	0.0009
seg1	0.09494	0.04140	152	2.29	0.0232
seg2	-0.07136	0.02565	152	-2.78	0.0061
CONDITION	0.3159	1.0816	48	0.29	0.7715
GENDER	2.1818	0.9988	48	2.18	0.0339
seg1*CONDITION	0.02260	0.05442	152	0.42	0.6786
seg2*CONDITION	0.04267	0.03274	152	1.30	0.1945
NEWAVO	-0.2481	2.1555	48	-0.12	0.9089
NEWANX	-0.1390	2.1514	48	-0.06	0.9488
neurcent	-1.8266	1.0421	48	-1.75	0.0860
NEWAVO*NEWANX	1.4045	2.0418	48	0.69	0.4948
seg1*NEWAVO	0.01095	0.06632	152	0.17	0.8691
seg1*NEWANX	-0.00905	0.03951	152	-0.23	0.8192
seg1*NEWAVO*NEWANX	-0.02240	0.04720	152	-0.47	0.6358
seg2*NEWAVO	-0.02620	0.04149	152	-0.63	0.5286
seg2*NEWANX	0.01425	0.02335	152	0.61	0.5426
seg2*NEWAVO*NEWANX	-0.00035	0.02757	152	-0.01	0.9899
CONDITION*NEWANX	3.5464	1.0217	48	3.47	0.0011
CONDITION*NEWAVO	-0.5755	1.4694	48	-0.39	0.6971
CONDIT*NEWAVO*NEWANX	1.7269	1.0994	48	1.57	0.1228
seg1*CONDITIO*NEWANX	0.06545	0.05172	152	1.27	0.2076
seg1*CONDITIO*NEWAVO	-0.06694	0.07565	152	-0.88	0.3776
seg1*COND*NEWA*NEWAN	0.004077	0.05525	152	0.07	0.9413
seg2*CONDITIO*NEWANX	-0.07633	0.03159	152	-2.42	0.0169
seg2*CONDITIO*NEWAVO	0.03371	0.04700	152	0.72	0.4743
seg2*COND*NEWA*NEWAN	-0.02070	0.03447	152	-0.60	0.5491
GENDER*NEWANX	-0.09940	1.1292	48	-0.09	0.9302
GENDER*NEWAVO	0.2103	1.1096	48	0.19	0.8505
GENDER*NEWAVO*NEWANX	-0.9982	0.9907	48	-1.01	0.3187

Model 1a: Splines with Attachment and neuro on Both Slopes split by condition

-- CONDITION=0 ----------------------------------

Solution for Fixed Effects

Effect	Estimate	Standard Error	DF	t Value	Pr > |t|
Intercept	9.8667	2.5627	21	3.85	0.0009
seg1	0.09520	0.03149	62	3.02	0.0036
seg2	-0.06827	0.01644	62	-4.15	0.0001
NEWAVO	0.3879	1.1451	21	0.34	0.7382
NEWANX	-0.4520	0.7246	21	-0.62	0.5395
neurcent	-1.0535	1.3664	21	-0.77	0.4493
NEWAVO*NEWANX	-0.2656	0.7964	21	-0.33	0.7421
seg1*NEWAVO	0.008065	0.05059	62	0.16	0.8739
seg1*NEWANX	-0.00664	0.02991	62	-0.22	0.8251
seg1*NEWAVO*NEWANX	-0.02456	0.03586	62	-0.68	0.4959
seg2*NEWAVO	-0.03250	0.02654	62	-1.22	0.2254
seg2*NEWANX	0.01211	0.01498	62	0.81	0.4221
seg2*NEWAVO*NEWANX	-0.00368	0.01757	62	-0.21	0.8347

Model 1b: Splines with Attachment and neuro on Both Slopes split by condition

-- CONDITION=1 ----------------------------------

Solution for Fixed Effects

Effect	Estimate	Standard Error	DF	t Value	Pr > |t|
Intercept	12.0815	3.0013	30	4.03	0.0004

seg1	0.1212	0.03840	90	3.16	0.0022
seg2	-0.03493	0.02604	90	-1.34	0.1831
NEWAVO	-0.4650	0.7851	30	-0.59	0.5581
NEWANX	3.4944	0.7380	30	4.73	<.0001
neurcent	-1.8367	1.4159	30	-1.30	0.2045
NEWAVO*NEWANX	1.3736	0.6274	30	2.19	0.0365
seg1*NEWAVO	-0.05363	0.03969	90	-1.35	0.1800
seg1*NEWANX	0.05671	0.03620	90	1.57	0.1207
seg1*NEWAVO*NEWANX	-0.01765	0.03128	90	-0.56	0.5741
seg2*NEWAVO	0.006262	0.02808	90	0.22	0.8240
seg2*NEWANX	-0.06089	0.02644	90	-2.30	0.0236
seg2*NEWAVO*NEWANX	-0.02259	0.02595	90	-0.87	0.3863

Split by gender

Model 1c: Splines with Attachment and neuro on Both Slopes split by gender

--- GENDER=1 ----------------------------------

Solution for Fixed Effects

Effect	Estimate	Standard Error	DF	t Value	Pr > |t|
Intercept	9.4908	1.9831	18	4.79	0.0001
seg1	0.000262	0.02578	54	0.01	0.9919
seg2	-0.02907	0.01706	54	-1.70	0.0942
NEWAVO	0.2652	0.6329	18	0.42	0.6802
NEWANX	0.9218	0.7068	18	1.30	0.2086
neurcent	-1.7912	1.0896	18	-1.64	0.1175
NEWAVO*NEWANX	3.4302	0.5684	18	6.03	<.0001
seg1*NEWAVO	0.004703	0.03091	54	0.15	0.8797
seg1*NEWANX	-0.06239	0.03386	54	-1.84	0.0709
seg1*NEWAVO*NEWANX	0.07102	0.02743	54	2.59	0.0123
seg2*NEWAVO	0.003351	0.02002	54	0.17	0.8677
seg2*NEWANX	0.002852	0.02479	54	0.12	0.9088
seg2*NEWAVO*NEWANX	-0.08871	0.02132	54	-4.16	0.0001

Model 1d: Splines with Attachment and neuro on Both Slopes split by gender

--- GENDER=2 ----------------------------------

Solution for Fixed Effects

Effect	Estimate	Error	DF	t Value	Standard Pr > |t|
Intercept	11.8768	3.2819	33	3.62	0.0010
seg1	0.1606	0.03605	98	4.46	<.0001
seg2	-0.05801	0.02445	98	-2.37	0.0196
NEWAVO	-0.3051	0.9999	33	-0.31	0.7622
NEWANX	1.5287	0.7469	33	2.05	0.0487
neurcent	-1.2132	1.5570	33	-0.78	0.4414
NEWAVO*NEWANX	-0.1692	0.7123	33	-0.24	0.8137
seg1*NEWAVO	-0.05999	0.04283	98	-1.40	0.1645
seg1*NEWANX	0.03199	0.03109	98	1.03	0.3061
seg1*NEWAVO*NEWANX	-0.05422	0.03155	98	-1.72	0.0888
seg2*NEWAVO	0.004528	0.03089	98	0.15	0.8838
seg2*NEWANX	-0.02413	0.02076	98	-1.16	0.2480
seg2*NEWAVO*NEWANX	0.007980	0.02268	98	0.35	0.7257
